# DNA Hypomethylation as a Potential Link between Excessive Alcohol Intake and Cardiometabolic Dysfunction in Morbidly Obese Adults

**DOI:** 10.3390/biomedicines10081954

**Published:** 2022-08-12

**Authors:** Imaduddin Mirza, Dina Naquiallah, Ariej Mohamed, Uzma Abdulbaseer, Chandra Hassan, Mario Masrur, Mohamed M. Ali, Shane A. Phillips, Abeer M. Mahmoud

**Affiliations:** 1 Division of Endocrinology, Diabetes and Metabolism, Department of Medicine, College of Medicine, The University of Illinois at Chicago, Chicago, IL 60612, USA; 2 Department of Surgery, College of Medicine, The University of Illinois at Chicago, Chicago, IL 60612, USA; 3 Department of Physical Therapy, College of Applied Health Sciences, The University of Illinois at Chicago, Chicago, IL 60612, USA; 4 Department of Kinesiology and Nutrition, College of Applied Health Sciences, The University of Illinois at Chicago, Chicago, IL 60612, USA

**Keywords:** alcohol, DNA methylation, obesity, cardiovascular, adipose tissues, inflammation, vasodilation, folate, B vitamins

## Abstract

A large percentage of obese patients in the United States suffer a comorbid substance use disorder, mainly alcohol use. Alcohol consumption interferes with the absorption of dietary methyl donors such as folate required for the one-carbon metabolism pathway and subsequently for DNA methylation. In this study, we assessed the association between alcohol consumption and DNA methylation in obese subjects. We obtained visceral adipose tissue (VAT) biopsies from bariatric patients. DNA methylation of 94 genes implicated in inflammation and immunity were analyzed in VAT in relation to alcohol consumption data obtained via questionnaires. Vasoreactivity was measured in the brachial artery and the VAT-isolated arterioles. Pro-inflammatory genes were significantly hypomethylated in the heavy drinking category correlating with higher levels of circulating inflammatory cytokines. Alcohol consumption correlated positively with body mass index (BMI), fat percentage, insulin resistance, impaired lipid profile, and systemic inflammation and negatively with plasma folate and vitamin B12, inflammatory gene DNA methylation, and vasoreactivity. In conclusion, these data suggest that alcohol intake is associated with lower DNA methylation and higher inflammation and cardiometabolic risk in obese individuals.

## 1. Introduction

A rising amount of epidemiological evidence suggests that excessive alcohol use is associated with an elevated risk of cardiovascular diseases (CVD) such as cardiomyopathy, hypertension, stroke, coronary artery diseases, and myocardial infarction [1]. This association depends mainly on the pattern of alcohol consumption. While mild and moderate alcohol drinking showed mixed effects on pathways related to vascular homeostasis, inflammation, and atherosclerosis, excessive and binge drinking is strongly associated with increased cardiovascular and metabolic risk [2]. Even for moderate drinking, adverse effects on mitochondrial function and redox homeostasis have been reported [1]. Two meta-analyses reported an association between moderate alcohol consumption (1–2 drinks/day for women and 2–3 drinks/day for men) and the risk of developing hypertension [3,4]. This association becomes more consistent and significant in subjects consuming greater levels of alcohol. Several mechanisms were reported to underlie these effects of alcohol on blood pressure, including its effect on inducing oxidative stress and vascular inflammation and subsequently impairing nitric oxide (NO) generation and bioavailability [5,6]. Furthermore, some studies reported an effect of alcohol in inducing vasoconstrictive pathways such as those mediated by prostanoids resulting in disturbances in the homeostasis of vasoactive mediators [7]. Our previous studies reported impairments in the endothelial-dependent vasodilation of the brachial artery and peripheral adipose tissue arterioles after a single bout of excessive (binge) drinking [8].

Obesity, a growing public health problem in the United States and worldwide, is associated with an increased risk of cardiovascular diseases, including hypertension, coronary heart disease, and stroke. This association contributes to obesity-related mortality in the United States, which rose substantially in the past 15 to 20 years [9]. There are obvious changes in the pattern of BMI distribution in the United States. These changes comprise a gradual move toward higher means of BMI due to high energy intake and sedentary behavior. More importantly, there is a striking increase in the upper range of the BMI distribution resulting in an expansion of a subgroup of morbidly obese individuals who are more vulnerable to the growing obesogenic environment in the community [10,11]. In support of this assumption, the number of annual bariatric surgeries rose from 70,256 in 2002 to 252,000 in 2018, according to the American Society for Metabolic and Bariatric Surgery [12]. Despite this accelerated growth in the number of morbidly obese patients, they are underrepresented in obesity-related research. We focused our investigation in the current study on the association between alcohol use and vascular dysfunction in a group of morbidly obese subjects scheduled for bariatric surgeries.

Some factors that may contribute to the increased susceptibility to morbid obesity are lack of impulse control and disturbances in neurochemical transmitters required to activate the reward system [13,14]. These neurobiological disorders are shared between substance use disorders and overeating-mediated obesity [15,16]. Thus, it is conceivable that those at risk of overeating problems are also at a higher risk of substance use disorders, including excessive alcohol intake, and are differentially impacted by the obesity epidemic. On the other hand, alcohol is a source of energy intake, and because 1 g of alcohol provides 7.1 kcal, alcohol consumption could contribute to weight gain if not compensated for [17]. Therefore, alcohol may have interlocking biological connections with obesity and cardiovascular diseases, yet its mediating role in obesity-related vascular dysfunction requires further investigation. Here, we study the differential intake of alcohol and its contribution to vascular dysfunction in morbidly obese individuals. We investigate the correlation between alcohol intake and flow-induced dilation (FID) in VAT-isolated arterioles. Finally, we examine the correlation between alcohol intake and several cardiometabolic risk factors, including levels of folate, vitamin B12, and homocysteine that we recently reported influencing microvascular function in morbidly obese individuals [18].

## 2. Materials and Methods

### 2.1. Human Participants

Eighty obese subjects (21 to 49 years old, BMI > 30 kg/m^2^) scheduled to have bariatric surgery at the University of Illinois Hospital were enrolled in this study. Women involved in the present study were premenopausal. Exclusion criteria included those above 50 years old, with a chronic or inflammatory illness that may alter vascular outcomes, pregnant women, smokers, and individuals with chronic heart, kidney, or liver diseases. Patients were informed about this research during their clinical visit, and those interested in participating were asked to provide written consent. The University of Illinois Institutional Review Board approved all forms and procedures used in this study following the most recent iteration of the *Declaration of Helsinki*. During the pre-surgery data collection visit at the University of Illinois Clinical Interface Core, blood samples, anthropometric/body composition measurements, and brachial artery ultrasound imaging were obtained. Questionnaires were used in order to obtain information about alcohol administration. Those who consumed alcohol were classified into three classes using criteria adapted from those published by Cahalan et al. [19]. We defined “mild drinkers” as those who had less than five drinks in one sitting less than once per month, those who had one to three drinks in one sitting less than three times per month, or two or fewer drinks in one sitting less than twice per week. “Moderate drinkers” were those who drank one to three times a month with three to four drinks at a time, those who drank one to two times a week with two to four drinks at a time, and those who drank three to six times per week only one drink each time. “Heavy drinkers” consumed alcohol at a higher quantity and frequency than moderate drinkers. Visceral adipose tissue (VAT) samples were collected from study subjects. Half of each sample was kept in cold HEPES buffer until dissection and isolation of microvessels. The second half of each sample was immediately frozen in liquid N_2_ for molecular analysis.

### 2.2. Anthropometric and Cardiometabolic Measurements

The subjects’ physical features, such as weight and BMI, were assessed. DXA (Dual X-ray absorptiometry) scan was utilized to measure total and visceral adipose tissue mass and muscle mass (iDXA, General Electric Inc., Boston, MA, USA). Fasting levels of plasma insulin and glucose were determined using our previously established procedures [18]. These values were used to compute the insulin resistance homeostasis model assessment (HOMA-IR) (fasting insulin (U/L) × fasting glucose (nmol/L)/22.5) [20]. As our prior publications detailed [18,21,22], total cholesterol, triglycerides, HDL, and LDL were evaluated using enzyme assays (Roche Diagnostics, Indianapolis, IN, USA). To measure hemoglobin A1c (HbA1c), we used the Crystal Chem SelfCheck kit (Elk Grove Village, IL, USA) and followed the manufacturer’s protocols.

The concentration of nitric oxide (NO) was indirectly measured in serum samples by detecting the NO metabolites (nitrate and nitrite) using the Nitrate/Nitrite Colorimetric Assay Kit (Cayman Chemicals, Ann Arbor, MI, USA) [23,24,25]. In brief, a nitrate reductase enzyme was used to convert all the nitrates to nitrites which were then converted to an azo molecule (dark purple) after adding the supplied Griess reagents 1 and 2. A multimode plate reader (Molecular Devices, San Jose, CA, USA) was then utilized to assess the absorbance at 540 nm as recommended by the manufacturer. CRP concentrations were determined using a high-sensitivity ELISA assay (Crystal Chem, Elk Grove Village, IL, USA) where samples, relevant controls, and provided standards (0.625–40 ng/mL) were applied to CRP antibody-coated plates, proceeded by working HRP solution, the substrate solution and finally the stop solution. Optical density was measured at 450 nm using a multimode plate reader. To measure interleukin 6 (IL-6) levels in plasma, High Sensitivity Magnetic Luminex Performance Assays were run according to the manufacturer’s guidelines (R&D). Samples were incubated with magnetic IL-6 antibody conjugated microparticles followed by biotinylated antibody and streptavidin–phycoerythrin conjugate. The microparticles were then reconstituted and analyzed utilizing the Luminex MAGPIX Instrument System (ThermoFisher Scientific, Waltham, MA, USA).

### 2.3. Plasma Homocysteine (Hcy) and One-Carbon Metabolism Factors

In order to collect measurements of Hcy concentration in plasma, a Hcy-specific ELISA assay (Cell Biolabs Inc., San Diego, CA, USA) was used [18]. Briefly, samples were incubated in the homocysteine-coated plates for ten minutes at RT, followed by incubation with the primary anti-homocysteine antibody and then the provided secondary antibody. Lastly, the substrate solution was added for 30 min, followed by the stop solutions. iMark Absorbance Microplate Reader (BioRad, Hercules, CA, USA) was used to read the absorbance of each sample at a wavelength of 450 nm. The Elecsys Folate III (Roche Diagnostics; Indianapolis, IN, USA) method was utilized to quantify folate and vitamin B12 concentrations using an intrinsic folate binding protein and vitamin B12 factor. Human Vitamin B6 (VB6) ELISA Kit (MyBioSource, San Diego, CA, USA) and Methionine Assay Kit (Fluorometric) (Abcam, Cambridge, MA, USA) were used to measure vitamin B6 and methionine, respectively, following the manufacturer’s protocols.

### 2.4. Flow-Mediated Dilation (FMD)

We measured brachial artery vasoreactivity using Hitachi Prosound Alpha 7 (Hitachi Aloka Medical America, Wallingford, CT, USA) [18]. First, the ultrasonography probe was positioned 1–3 cm proximal to the ante-cubital fossa with a 60-degree tilt. A blood pressure measuring cuff was inflated to 220 mmHg around the mid-forearm for five minutes. A video was recorded for the baseline (BSL) arterial diameter (60 s before cuff inflation) and 300 s after cuff deflation (reactive hyperemia (RH)). The Automated Edge Detection software system was used to capture images for subsequent analysis. As previously reported, relative FMD was computed using the highest brachial artery diameter at BSL minus the largest mean measures recorded after cuff deflation [percent FMD = (RH diameter in mm − BSL diameter in mm/BSL diameter in mm × 100)] [18,21,24,25,26]. Applanation tonometry (Millar Instruments, Houston, TX, USA) was used to measure central pulse wave velocity from the waveform at the carotid and femoral (central) site using the SphygmoCor software (AtCor Medical, Sydney, Australia).

### 2.5. Measurements of Microvascular Flow-Induced Dilation

Adipose tissue samples were dissected to isolate resistant arterioles, which were then purged of extra connective tissue and fat. As we previously reported, the internal diameter of dissected vessels was measured in response to a gradually increasing pressure gradient [21,23,24,25,27]. Vessels were cannulated using glass microcapillaries inserted into an organ perfusion chamber. The vascular ends were tied around the microcapillaries with nylon Ethilon monofilament. The organ chamber was then transferred to an inverted microscope connected to video microscopy to monitor arteriolar vasoreactivity. Terminals of the cannulated arterioles were connected to two reservoirs containing Krebs buffer. Moving the reservoirs in equal and opposite directions (up and down) created an intraluminal pressure gradient (10–100 cm H_2_O). The Krebs buffer solution was supplemented with a mixture of O_2_ (21%), CO_2_ (5%), and N_2_ (74%), and its pH and temperature were maintained at 7.4 and 37 degrees Celsius, respectively. Following baseline measurement, cannulated vessels were constricted with angiotensin II (10^−6^ mol/L) (Sigma Aldrich, St. Louis, MO, USA), and only those constricted more than 30% were used for measurements. Arteriolar vasoreactivity was measured in the presence and absence of the endothelial nitric oxide synthase inhibitor, L-N^G^-Nitro arginine methyl ester (L-NAME, 10^−4^ mol/L). The percentage of vasodilation was calculated by normalizing arteriolar diameter in each pressure gradient to that after angiotensin II.

### 2.6. Methylation PCR Analysis

Promoter methylation was analyzed in a panel of 94 genes related to inflammation and immunity using EpiTect Methyl II PCR Arrays (Qiagen, Chatsworth, CA, USA). In order to avoid cell heterogeneity, visceral adipose tissue (VAT) was enzymatically digested with 0.1% collagenase type I, and mature adipocytes were isolated following previously published protocol [28,29]. DNA was extracted from visceral adipocytes via DNeasy Blood and Tissue kit (Qiagen), and its quantity and quality were tested via UV-Vis Spectrophotometer (Thermo Fisher Scientific, Waltham, MA, USA). Acceptable purity was determined by the A260/A230 and A260/A280 ratios (>1.7 and >1.8, respectively). Extracted DNA was processed via the supplied kit-provided restriction enzymes. In this reaction, mixtures of the extracted DNA (2 µg) and the methylation-dependent (Md) and/or methylation-sensitive (Ms) enzymes were prepared and heated for 6 h at 37 °C, then heat-inactivated for 20 min at 65 °C. PCR arrays were then performed using ViiA 7 Real-Time PCR System (Thermo Fisher Scientific) and the manufacturer’s PCR cycling recommendations. An excel sheet supported by the MethylScreenTM technology was provided by Qiagen to automatically calculate the percentage of DNA methylation for each gene in the array using Ct values.

### 2.7. Real-Time PCR (Polymerase Chain Reaction)

Total RNA was extracted from the adipose tissue samples via RNeasy mini kits (Qiagen, Germantown, MD, USA). The quality and quantity of extracted RNA were tested via UV-Vis Spectrophotometer (Thermo Fisher Scientific, Waltham, MA, USA). cDNA was generated via reverse transcription of the RNA using iScript™ Supermix, and real-time PCR assays were performed using SYBR Green Supermix (Biorad Laboratories, Hercules, CA, USA). PCR primers were designed via the online software primer3 (Supplementary Table S1) and constructed by Invitrogen Life Technologies. The housekeeping gene, GAPDH, was used to normalize gene expression calculated using the Livak method (2^−∆∆Ct^) [30].

### 2.8. Statistical Analyses

Data were presented as mean ± standard error (SE) and were considered statistically significant when the *p*-value was less than 0.05. To compare variables among the different alcohol consumption categories, we used one-way ANOVA and post hoc test whenever appropriate. Bonferroni correction method was used to calculate the adjusted *p*-value for multiple comparisons (*p* < 0.0004) for gene methylation and expression data. The methylation percentage of differentially methylated genes was averaged to calculate the methylation score. A bivariate Pearson Correlation and χ^2^ tests were used to test the correlation between continuous variables and categorical variables, respectively. Multiple regression analysis was used to determine the independent association between alcohol consumption and cardiometabolic risk factors. In model 1, we adjusted for age, race, and gender; in model 2, we adjusted for age, race, gender, and BMI. The continuous variables were dichotomized using the median value, and the no/mild drinker group was used as a reference group. Analyses were performed using SPSS statistical package version 28 (SPSS Inc., Chicago, IL, USA).

## 3. Results

### 3.1. Physical Characteristics and Cardiometabolic Risk Factors

Anthropometric measurements and cardiometabolic risk factors were summarized in Table 1. Body weight and BMI were considerably greater in the moderate and heavy alcohol consumption groups compared to the no/mild alcohol drinking group. DEXA-measured percentages of total and visceral fat rose with increased alcohol use, with heavy drinkers having the greatest percentages (*p* < 0.0001). Table 1 also displays cardiometabolic risk factors such as heart rate, blood pressure, and lipid and glucose metabolism measurements. Heavy drinkers had considerably higher systolic and diastolic blood pressure levels than the other two groups. Fasting levels of blood glucose, insulin resistance index (HOMA-IR), total cholesterol, triglycerides, and LDL were significantly greater in heavy drinkers than in mild/no and moderate drinkers. At the same time, HDL was significantly lower in the former group (*p* < 0.0001). Similarly, the systemic inflammation markers, interleukin 6 (IL-6), and C-reactive protein (CRP) were found to be higher in the plasma of heavy drinkers compared to two other groups. 

There were no significant differences in metabolic profiles or systemic inflammation markers between no/mild and moderate drinkers. Nonetheless, measures of vascular function consistently decreased as alcohol use went from no/mild to moderate to heavy. When compared to the no/mild drinking group, the average brachial artery flow-mediated dilation (FMD) was 30% and 60% lower in the moderate and heavy drinkers, respectively (*p* < 0.0001). Similarly, the average baseline FID in VAT-isolated arterioles was 28% and 69% lower in moderate and heavy drinkers than in no/mild drinkers. Furthermore, the FID dependence on nitric oxide (NO) was measured via arteriolar incubation with the eNOS inhibitor, L-NAME. Our results showed that arteriolar FID was less dependent on NO in moderate and heavy drinkers compared to no/mild drinkers, which was consistent with the considerable drop in systemic NO bioavailability in these groups. Finally, as alcohol consumption increased, pulse wave velocity (PWV) increased dramatically, indicating arterial stiffness.

### 3.2. DNA Methylation and Expression of Inflammatory Genes

One-carbon metabolism (folate cycle) plays a crucial role in biological methylation processes, including DNA methylation. In this cycle, nutrients such as folate, vitamin B6, vitamin B12, and methionine participate in the synthesis of S-adenosyl methionine, which is the universal methyl donor for DNA methylation. Several studies showed that even at moderate levels of intake, alcohol might interfere with methyl donor absorption from the intestine, uptake by the liver, and conversion by the kidneys [31]. In this study, we measured these nutrients in relation to alcohol consumption. Average folate levels were 27% lower in moderate drinkers and 51% lower in heavy drinkers compared to no/mild drinkers (*p* < 0.0001). Similarly, plasma levels of vitamins B12 and B6 declined as alcohol consumption rose from mild to moderate to heavy, while methionine showed no significant variations (Table 2).

S-adenosyl methionine is metabolized to SAH (S-adenosylhomocysteine); the latter can be reversibly transformed to homocysteine (Hcy) by the SAH hydrolase enzyme. Several studies have reported an association between hyperhomocysteinemia and aberrant DNA methylation. Furthermore, high concentrations of Hcy were found to be an independent risk factor for cardiovascular diseases. Some studies have claimed that alcohol raises blood Hcy levels due to its adverse effects on folate and vitamin B12 [32,33]. In this study, levels of Hcy were considerably higher in the heavy drinkers compared to other alcohol drinking categories. Hcy levels were 25% higher on average in moderate drinkers, but this difference did not achieve statistical significance due to sizeable interpersonal variability (Table 2).

Promoter methylation was analyzed in 94 genes implicated in inflammation and immune function (Supplementary Table S2). When averaged, the percentage of promoter methylation across the gene array was 71% lower in moderate drinkers and 106% lower in heavy drinkers compared to no/mild drinkers (*p* < 0.0001). Nearly 68% of the array genes, mostly pro-inflammatory genes, exhibited promoter hypomethylation in the higher drinking categories (moderate and heavy) relative to no/mild drinkers. After multiple testing adjustments, 18 genes in the PCR array showed remarkable differences in DNA methylation of pro-inflammatory genes among alcohol drinking categories. Table 2 summarizes the percentage of promoter methylation in these genes in each group. To test the effect of this methylation profile on gene transcription, we measured the mRNA expression of the 18 genes via real-time PCR. Ten of the 18 differentially methylated pro-inflammatory genes showed concomitant increases in mRNA levels and were differentially expressed among alcohol drinking categories. On the top of these genes are HDAC5, NFκB, and TNFRSF8 (*p* < 1 × 10^−10^).

Employing logistic regression models with adjustments for age, race, and gender (model 1) or age, race, gender, and BMI (model 2), we found that alcohol intake was significantly associated with the likelihood of cardiovascular risk factors (Table 3). For hypertension, diabetes, and dyslipidemia, medical history or standard cutoffs published by the AHA (American Heart Association) or the ADA (American Diabetes Association) were used for classification. For the rest of the variables included in Table 3, the median values were used to dichotomize continuous variables, and the no/mild category was used as the reference. To summarize DNA methylation data, a methylation score was estimated by calculating the average methylation of pro-inflammatory genes that were differentially methylated. In model 2, where BMI was accounted for, moderate drinkers were found to have a 20% higher risk of hypertension, a 16% higher risk of homocysteinemia, a 19% higher risk of elevated systemic inflammation, an 11% higher risk of impaired brachial artery FMD, a 45% higher risk of impaired arteriolar FID, an 18% higher risk of one-carbon metabolism nutrient deficiency, and a 60% higher risk of low methylation score. Among heavy drinkers, the risk of hypertension increased by 33%, the risk of dyslipidemia increased by 29%, the risk of homocysteinemia increased by 67%, the risk of systemic inflammation increased by 31%, the risk of impaired arterial FMD increased by 41%, the risk of impaired arteriolar FID increased by 73%, the risk of arterial stiffness increased by 16%, the risk of one-carbon metabolism nutrient deficiency increased by 81%, and the risk low methylation score increased by 68%.

## 4. Discussion

Epigenetic modifications have been shown to reflect the body’s exposure to various lifestyle factors such as nutrition, physical activity, and alcohol consumption [34]. As a result, these epigenetic alterations and their relevance to cardiovascular and metabolic morbidities are of considerable interest. Excessive alcohol intake is a prevalent lifestyle factor linked to cardiometabolic diseases such as hypertension, dyslipidemia, myocardial infarction, and coronary artery disease; hence restricted alcohol intake is commonly recommended for managing these diseases [1].

Alcohol is expected to interfere with the absorption and bioavailability of the one-carbon cycle nutrients known to mediate DNA methylation, such as folate and other B vitamins [31]. Therefore, excess alcohol intake is expected to induce aberrant DNA methylation patterns, mostly hypomethylation. However, a few studies investigated these patterns in circulating blood cells yielding inconsistent findings [35,36,37,38,39]. Moreover, it is unclear how these patterns relate to specific metabolic or vascular characteristics in underrepresented morbidly obese persons. To this end, we investigated differential methylation profiles of inflammatory genes in VAT of morbidly obese adults classified into three categories based on their alcohol intake. Furthermore, this cohort used regression models to estimate the association between alcohol intake and cardiometabolic risk factors, and vascular function. 

Recently, our group reported a critical role of VAT-expressed inflammatory genes as a source of systemic inflammation in morbidly obese individuals [22]. We also reported a significant association between the expression of these genes and impaired cardiometabolic functions. Therefore, in the current study, we sought to analyze the DNA methylation patterns of VAT inflammatory genes under different levels of alcohol consumption and whether they are associated with the differential expression of those genes. The major results of the current investigation are that alcohol consumption is associated, in a dose-dependent manner, with (1) impaired metabolic and vascular function; (2) lower levels of methyl donor nutrients, mainly folate and vitamins B6 and B12; (3) DNA hypomethylation and increased expression of VAT inflammation-related genes; (4) induced systemic inflammation; and (5) increased risk of hypertension, dyslipidemia, homocysteinemia, and arterial stiffness in morbidly obese adults after adjusting for BMI.

Previous studies on the relationship between alcohol use and blood pressure produced contradictory results. Some of these studies reported null associations; some found a U-shaped link between alcohol consumption and the risk of hypertension, while others indicated an increased risk of hypertension with moderate and excessive alcohol consumption [40]. It is worth mentioning that most of the studies that reported null associations were conducted in young, healthy participants who might not be fully representative of the whole population. In the Kaiser Permanente Study, Klatsky et al. [41] reported a dose-dependent relationship between alcohol intake and blood pressure in White Americans, with the highest blood pressure values reported for those who consumed 6–8 drinks per day. This relationship was more evident in White men and persons aged 55 years or older. In the Atherosclerosis Risk in Communities Study, consuming ≥ 210 g of alcohol per week by men aged 45 to 64 was an independent risk factor for hypertension [42]. On the other hand, in the Physicians’ Health Study [43], mild-to-moderate alcohol consumption was associated with decreased hypertension risk in women and increased risk in men. Finally, a meta-analysis by Corrao et al. [44] that included 156 studies and nearly 117,000 subjects demonstrated an association between any level of alcohol intake and increased risk of hypertension. In our current diversified study, the observed association between increased alcohol intake and the risk of hypertension was dose-dependent and persisted after adjusting for confounding factors such as age, gender, and race. Conceivably, studies with diverse populations can better detect the relationship between alcohol use and hypertension risk. As a result, further research, including a more diverse population sample and studies that consider various lifestyle factors and health status, is needed to better understand the relationship between alcohol intake and cardiovascular risk.

Dose-response associations have also been reported between alcohol and circulating lipids such as LDL, HDL, and triglycerides [45,46]. Some studies have indicated a protective effect of moderate alcohol consumption manifested as elevated HDL levels. This action is thought to be mediated by inhibiting cholesteryl-ester transfer protein (CEPT), which is responsible for moving cholesterol from HDL to LDL particles and increasing HDL concentrations [47]. Heavy drinking, on the other hand, has been linked to greater triglyceride levels due to alcohol-induced inhibition of lipoprotein lipase and accelerated production of VLDL (very low-density lipoprotein) [48]. Nevertheless, inconsistent findings regarding the relationship between alcohol and lipid profiles exist. For example, a study on 6912 Polish men demonstrated that the risk of hypertriglyceridemia increased by 25% in moderate alcohol drinkers versus 46% in heavy drinkers [49]. Finally, a meta-analysis of 63 studies (1686 subjects) evaluating the relationship between alcohol and dyslipidemia demonstrated a dose-dependent increase in HDL and a lack of significant changes in total cholesterol, LDL, and triglycerides. In this study, significant increases in triglycerides were only observed at the highest dose of alcohol intake (>60 g/day) [50]. In consistency with these findings, we observed an increased risk of dyslipidemia only in the heavy drinking category following adjustment for BMI (model 2) in the current study.

The association between alcohol consumption and diabetes risk is unclear, and counterintuitive findings of greater rates of diabetic patients among abstainers and light drinkers have been reported in French [51], Danish [52], and US populations [43,53]. These findings may be attributed to diabetes patients’ tendency to cease or minimize drinking alcohol as prescribed by their doctors. Our findings showed a lack of any significant association between alcohol intake and the likelihood of diabetes after adjusting for BMI.

Alcohol’s interaction with folic acid/homocysteine or one-carbon metabolism is one of the processes underpinning its toxicity. One-carbon metabolism is a key source of methyl groups for DNA methylation and subsequently for regulating gene transcription. In our study, heavy alcohol consumption was associated with a higher risk of homocysteinemia, and plasma Hcy levels were significantly higher in heavy drinkers compared with mild and moderate drinkers. A previous study by Bleich et al. [33] reported that daily consumption of 30 g of alcohol for six weeks increased Hcy levels by 17–25% in 60 healthy German men. A study by Sakuta et al. [54] on Japanese men aged 51 to 59 reported hyperhomocysteinemia among alcohol drinkers compared to abstainers. Contradictory findings were reported by Burger et al. [55], where an inverse association between Hcy concentrations and alcohol consumption was observed in 2365 German men between 18 and 79 years old. According to the findings of Burger’s study, 75% of the alcohol consumed by this population came from beer, a vitamin B source that may help lower Hcy levels. Accordingly, future research must take into account the type of beverage in their evaluations.

In addition to homocysteinemia, alcohol intake in our study showed a significant correlation with systemic inflammation and decreased nitric oxide bioavailability, all of which are risk factors for vascular dysfunction. Thus, in the current study, we measured the association between alcohol intake and vascular function assessed in vivo in medium-sized arteries and ex vivo in isolated VAT arterioles. The association between increased alcohol consumption and impaired vascular function was dose-dependent and robust even after adjusting for confounding variables, including the BMI. In contrast to previous findings in healthy individuals, a biphasic U- or J-shaped association between alcohol use and cardiometabolic risk was not detected in the current morbidly obese cohort. It is worth mentioning that in two recent meta-analyses, a dose-dependent linear association was shown between alcohol intake and the risk of coronary artery disease, hypertension, stroke, and heart failure, which contradicts the protective effect of low to moderate alcohol use [56,57]. In addition, recent studies have revealed disparities in the association between moderate alcohol consumption and cardiovascular risk among people of diverse racial/ethnic backgrounds or health conditions [58,59,60], making it difficult to generalize the protective effect of moderate alcohol consumption to all individuals.

Alcohol intake, both moderate and heavy, predicted lower levels of folate cycle nutrients (i.e., methyl donors and cofactors) such as folate, vitamin B6, and vitamin B12. The folate (one-carbon) cycle is a chemical reaction where methyl groups are transferred to DNA nucleotides resulting in DNA methylation [61]. Therefore, it is probable that molecular mechanisms underlying the link between alcohol use and cardiometabolic dysfunction involve abnormal DNA methylation patterns. Informed by our prior research demonstrating that VAT is a key source of inflammation in obese individuals [22], we tested the hypothesis that alcohol is associated with an aberrant DNA methylation and, consequently, an upregulation of inflammation-related genes in VAT. Indeed, our results indicated significant reductions in the promoter methylation of 18 pro-inflammatory genes in VAT as alcohol intake increased from mild to moderate to heavy. This effect also accompanied augmented mRNA expression in 10 of those genes. Furthermore, moderate and heavy alcohol consumption was associated with 60% and 68% increases in the likelihood of having a reduced methylation score, respectively.

Overall, the present study provides a unique set of data, suggesting an underlying epigenetic mechanism for alcohol-induced inflammation, vascular dysfunction, and subsequently increased cardiometabolic risk in morbidly obese individuals. According to our findings and those of prior epigenome-wide association studies, DNA methylation may serve as a novel biomarker for alcohol intake and cardiometabolic risk. For instance, in an epigenome-wide association study by Wilson et al. [62], increased alcohol consumption by 2878 non-Hispanic White women was associated with significantly lower average blood DNA methylation across an entire CpG array (450K CpGs). Sites with decreased methylation were enriched for genes involved in inflammatory cell recruitment, insulin and corticosteroid signaling, and endothelial cell function. Another epigenome-wide association study by Liu et al. [63] investigated blood DNA methylation in relation to alcohol intake in 13,317 participants from a diverse racial/ethnic background (54% women; aged 42–76 years). They identified 144 CpGs from whole blood DNA samples and 62 CpGs from monocyte-derived DNA that provided robust predictions for current heavy alcohol consumption (≥42 and ≥28 g per day in men and women, respectively). The differential methylation in this cohort was associated with upregulated expression levels of genes involved in inflammation and immune function.

The role of DNA methylation as a predictive marker and a therapeutic target in cardiovascular diseases was highlighted in the systematic review/meta-analysis by Palou-Márquez et al. [64] where DNA methylation data from the Framingham Offspring Study were integrated with gene expression data to identify biomarkers of cardiovascular risk. This multi-omics data integration approach identified several DNA methylation patterns in genes involved in inflammation, endothelium homeostasis, and cardiac remodeling that were key players in determining cardiovascular risk. Overall, the findings of this study and earlier research point to DNA methylation as a possible link between excessive alcohol intake and cardiometabolic risk factors. Nonetheless, further research is needed to examine this association in different populations with varying age groups, racial/ethnic backgrounds, comorbidities, and lifestyles. Tobacco smoking ranks first among these lifestyle variables since multiple behavioral research have reported a substantial link between alcohol use and smoking [65]. We excluded smokers from the current study because we aimed to investigate the association between alcohol and cardiometabolic risk that was not confounded by smoking. Future research is needed to examine any potential synergism between alcohol use and smoking in promoting aberrant DNA methylation and cardiometabolic risk.

Several positive aspects can be attributed to this study. First is the inclusion of a diverse cohort of male and female subjects from different ages and racial backgrounds. Furthermore, instead of easily accessible blood samples that provided conflicting results in prior studies, the researchers tested DNA methylation of a large group of inflammation-related genes in surgically acquired VAT from morbidly obese people. Additionally, the functional consequences of the observed methylation profiles were assessed by analyzing the mRNA expression of the differentially methylated genes. Moreover, alcohol intake was examined for its association with a huge set of crucial cardiometabolic risk variables, including DNA methylation and expression of inflammatory genes, one-carbon metabolism factors (folate and vitamin B12), and in vivo and ex vivo measured vascular function, among other metabolic and vascular biomarkers. Finally, in contrast to earlier research that only assessed macrovessels such as the femoral and brachial arteries, we studied the vasoreactivity of microvessels, which are expected to be influenced promptly by molecular changes in the VAT. Furthermore, because smaller arterioles are the principal source of peripheral vascular resistance and regulation of blood pressure, the present study is translationally meaningful.

This study does, however, have certain drawbacks. First, the small sample size has reduced the capacity to identify significant changes in many genes after controlling for multiple testing. The small sample size may also have diminished the association between alcohol consumption and many cardiometabolic risk variables investigated. The adoption of a narrow array of preselected genes, despite serving the objective of targeting inflammation-related mechanisms efficiently, restricts the capacity to explore potentially significant pathways among the alcohol drinking categories. Therefore, it is imperative that future research examine DNA methylation using broader DNA methylation arrays. Finally, the current research is cross-sectional in nature. As a result, drawing causative conclusions or prescribing a particular direction for the link between alcohol use and cardiometabolic risk in obese patients is improper. Accordingly, more longitudinal and interventional research is needed to better understand the nature of this relationship.

## Figures and Tables

**Table 1:** Subject characteristics and cardiometabolic risk factors.

Variable	No/Mild (*n* = 33)	Moderate (*n* = 27)	Heavy (*n* = 20)	*p*-Value
Age, y	37 ± 7	36 ± 8	33 ± 8	0.2714
Gender (♀)	19	18	13	0.7427 ¥
Race/ethnicity (AA)	16	13	9	0.1261
**Anthropometric DEXA measurements**				
Weight, kg	105.7 ± 31.9	121.3 ± 32.9	143.9 ± 29.3 *	0.0003
WC, cm	112.2 ± 21.1	131.6 ± 14.8 *	139.3 ± 44.9 *	0.0015
BMI, kg/m^2^	37.5 ± 11.3	43.3 ± 11.5	46.7 ± 7.7 *	0.0082
Fat %	45.6 ± 11.2	51.6 ± 2.5 *	58.1 ± 2.8 *†	<0.0001
Lean %	52.8 ± 10.5	47.2 ± 2.4 *	41.0 ± 2.9 *†	<0.0001
Trunk fat %	48.2 ± 11.8	55.8 ± 3.8 *	60.2 ± 3.8 *	0.0158
VAT mass, kg	1.4 ± 0.2	1.6 ± 0.2 *	2.6 ± 0.2 *†	<0.0001
**Metabolic and cardiovascular measurements**				
FPI, µU/mL	12.7 ± 4.5	14.81 ± 4.5	16.04 ± 2.3 *	0.0127
FPG, mg/dL	96.7 ± 12.3	99.18 ± 9.7	112.35 ± 15.9 *†	0.0001
HOMA-IR	3.2 ± 1.8	3.8 ± 1.8	5.4 ± 0.8 *†	0.0001
HbA1c, %	5.4 ± 0.2	5.8 ± 0.3	5.9 ± 1.3 *	0.0203
Chol, mg/dL	166.5 ± 29.0	163.3 ± 28.3	186.8 ± 22.0 *†	0.0097
LDL, mg/dL	97.1 ± 17.0	97.4 ± 27.3	13.5 ± 21.9 *†	0.0203
HDL, mg/dL	45.0 ± 11.7	44.8 ± 13.5	37.0 ± 6.7 *	0.0305
Trig, mg/dL	105.7 ± 25.7	111.6 ± 19.8	141.0 ± 16.7 *†	<0.0001
HR, bpm	78 ± 14	77 ± 9	83 ± 6	0.1484
SBP, mmHg	124 ± 16	127 ± 17	140± 16 *†	0.0036
DBP, mmHg	78 ± 9	78 ± 10	87 ± 11 *†	0.0035
Brachial FMD, %	10.9 ± 4.1	8.2 ± 2.0 *	5.9 ± 2.7 *†	<0.0001
PWV, ms-1	9.5 ± 1.1	10.3 ± 1.2 *	12.4 ± 0.8 *†	<0.0001
Baseline FID, %	48.7 ± 19.8	36.9 ± 16.1 *	23.8 ± 1.6 *†	<0.0001
L-NAME ∆ FID, %	80.7 ± 25.8	53.1 ± 19.1 *	25.9 ± 10.2 *†	<0.0001
Serum NO, µmol/L	5.5 ± 0.7	4.5 ± 1.2 *	3.6 ± 1.0 *†	<0.0001
**Circulating biomarkers of inflammation**				
CRP, mg/dL	2.3 ± 1.7	3.1 ± 1.8	4.8 ± 1.4 *†	<0.0001
IL6, pg/mL	13.4 ± 6.1	13.3 ± 12.9	27.1 ± 11.9 *†	<0.0001

* For significant difference compared to the No/Mild group using One-Way ANOVA; † for significant difference comparing the Severe and Moderate groups using One-Way ANOVA; ¥ chi-square test was used to compare the gender among groups; AA refers to participants from an African American heritage versus Caucasians. FID % reflects the measurements at ∆ 60 cmH2O (physiological pressure gradient). L-NAME ∆ FID is the % reduction in FID after L-NAME relative to baseline FID. BMI, body mass index; Chol, cholesterol; CRP, C-reactive protein; DBP, diastolic blood pressure; FPG, fasting plasma glucose; FPI, fasting plasma insulin; HbA1c, glycosylated hemoglobin; HDL, high-density lipoprotein; Hcy, HOMA-IR, homeostatic model assessment for insulin resistance; HR, heart rate; IL6, interleukin 6; homocysteine; LDL, low-density lipoprotein; NO, nitric oxide; SBP, systolic blood pressure; PWV, pulse wave velocity; Trig, triglycerides; VAT, visceral adipose tissues; WC, waist circumference.

**Table 2:** DNA methylation and expression of inflammatory genes.

Variable	No/Mild (*n* = 33)	Moderate (*n* = 27)	Heavy (*n* = 20)	*p*-Value
**One-carbon metabolism factors**				
Folate, ng/mL	22.5 ± 4.8	17.2 ± 4.9 *	13.4 ± 6.2 *†	<0.0001
Vit B12, ng/L	488.7 ± 181.8	308.2 ± 150.9 *	294.2 ± 168.1 *	<0.0001
Vit B6, μg/L	40.1 ± 7.9	33.1 ± 8.7 *	36.1 ± 6.3	0.0037
Methionine, µmol/L	37.7 ± 9.1	33.9 ± 10.2	34.8 ± 11.5	0.3210
Hcy, µmol/L	10.3 ± 4.2	13.3 ± 6.3	21.5 ± 9.2 *†	<0.0001
**DNA methylation of inflammatory genes in VAT (%)**				
CXCL1	57.5 ± 40.8	9.2 ± 20.0 *	9.8 ± 14.8 *	<0.0001
CXCR2	55.4 ± 25.5	45.3 ± 11.6	29.5 ± 11.1 *†	<0.0001
HDAC5	85.9 ± 17.6	68.8 ± 14.9 *	15.5 ± 3.8 *†	<0.0001
IGFBP3	93.9 ± 34.1	89.1 ± 22.8	48.9 ± 13.8 *†	<0.0001
IL12RB2	84.4 ± 15.7	22.3 ± 9.5 *	17.2 ± 11.5 *	<0.0001
IL1R1	88.3 ± 18.9	52.3 ± 19.1 *	33.4 ± 17.4 *†	<0.0001
IL7	93.0 ± 19.5	86.3 ± 18.7	22.8 ± 8.1 *†	<0.0001
IL12A	41.9 ± 44.7	14.5 ± 8.9 *	10.5 ± 12.1 *	0.0002
IL17RA	37.3 ± 35.9	12.8 ± 3.5 *	9.2 ± 6.3 *	0.0001
MYD88	69.5 ± 33.4	25.4 ± 11.6 *	25.5 ± 5.5 *	<0.0001
NFATC3	47.4 ± 13.8	44.7 ± 16.5	32.1 ± 10.7*†	0.0005
NFκB	94.5 ± 14.1	53.7 ± 27.2 *	55.9 ± 19.5 *	<0.0001
NFKBIB	54.8 ± 41.9	20.0 ± 12.4 *	12.7 ± 21.4 *	<0.0001
SMAD3	95.2 ± 15.2	69.1 ± 11.5 *	57.5 ± 19.1 *†	<0.0001
TGFBR2	94.9 ± 24.4	26.8 ± 19.6 *	16.7 ± 11.5 *	<0.0001
TLR5	35.7 ± 16.6	17.9 ± 7.6 *	5.6 ± 4.1 *†	<0.0001
TNFRSF8	31.1 ± 9.3	25.1 ± 7.5 *	22.6 ± 5.3 *	0.0005
TRAF6	80.0 ± 25.2	81.5 ± 17.5	50.5 ± 22.3 *†	<0.0001
**Differentially expressed inflammatory genes in VAT (fold change ‡)**				
CXCL1	1.0 ± 0.4	1.7 ± 0.2 *	1.9 ± 0.3 *	<0.0001
CXCR2	1.0 ± 0.2	1.6 ± 0.1 *	2.3 ± 0.2 *†	<0.0001
HDAC5	1.0 ± 0.1	1.4 ± 0.1 *	1.8 ± 0.3 *†	<0.0001
IGFBP3	1.0 ± 0.1	1.3 ± 0.3 *	2.4 ± 0.1 *†	<0.0001
IL12RB2	1.0 ± 0.3	1.9 ± 0.1 *	2.0 ± 0.1 *	<0.0001
IL17RA	1.0 ± 0.1	1.1 ± 0.3	1.3 ± 0.4 *†	0.0004
NFATC3	1.0 ± 0.1	0.9 ± 0.2	1.8 ± 0.2 *†	<0.0001
NFκB	1.0 ± 0.3	1.7 ± 0.1 *	1.5 ± 0.4 *	<0.0001
TGFBR2	1.0 ± 0.3	2.7 ± 0.4 *	3.0 ± 0.6 *	<0.0001
TNFRSF8	1.0 ± 0.2	2.0 ± 0.4 *	2.2 ± 0.2 *	<0.0001

* For significant difference compared to the No/Mild group using One-Way ANOVA; † for significant difference comparing the Severe and Moderate groups using One-Way ANOVA; ‡ No/Mild is the reference group for fold change calculations.

**Table 3:** Likelihood of cardiometabolic risk factors in relation to alcohol consumption levels.

Variable	No/Mild (*n* = 33)	Moderate (*n* = 27)	Sig	Heavy (*n* = 20)	*p*-Value
**Hypertension**					
Model 1	1	1.37 (1.08−1.62)	0.0024	1.49 (1.20−1.64)	<0.0001
Model 2	1	1.20 (0.95−1.35)	0.0416	1.33 (1.12−1.56)	0.0008
**Diabetes**					
Model 1	1	1.11 (0.93−1.19)	0.0967	1.32 (1.13−1.47)	<0.0001
Model 2	1	1.01 (0.93−1.09)	0.8177	1.07 (0.91−1.25)	0.4109
**Dyslipidemia**					
Model 1	1	1.22 (1.03−1.48)	0.0313	1.42 (1.15−1.67)	0.0002
Model 2	1	1.05 (0.96−1.08)	0.1042	1.29 (1.09−1.53)	0.0033
**Homocysteinemia**					
Model 1	1	1.34 (1.17−1.72)	0.0003	1.61 (1.22−1.81)	<0.0001
Model 2	1	1.16 (1.01−1.37)	0.0559	1.67(1.09−1.88)	0.0002
**Systemic inflammation**					
Model 1	1	1.27 (0.98−1.43)	0.0131	1.39 (1.16−1.55)	<0.0001
Model 2	1	1.19 (1.01−1.33)	0.0132	1.31 (1.01−1.51)	0.0085
**Arterial FMD**					
Model 1	1	1.29 (1.09−1.64)	0.0145	1.54 (1.22−1.84)	<0.0001
Model 2	1	1.11 (1.08−1.26)	0.0079	1.41 (1.18−1.77)	0.0009
**Arterial stiffness**					
Model 1	1	1.17 (0.97−1.35)	0.0622	1.19 (0.96−1.34)	0.0405
Model 2	1	1.09 (1.01−1.29)	0.1682	1.16 (0.92−1.23)	0.0448
**Arteriolar FID**					
Model 1	1	1.67 (1.37−1.91)	<0.0001	1.75 (1.62−1.95)	<0.0001
Model 2	1	1.45 (1.24−1.75)	<0.0001	1.73 (1.52−1.93)	<0.0001
**One-Carbon metabolism factors**					
Model 1	1	1.22 (1.03−1.44)	0.0198	1.78 (1.27−1.90)	<0.0001
Model 2	1	1.18 (1.08−1.36)	0.0049	1.81 (1.51−1.95)	<0.0001
**Inflammatory gene methylation score**					
Model 1	1	1.29 (1.20−1.57)	<0.0001	1.34 (0.99−1.52)	0.0197
Model 2	1	1.60 (1.31−1.82)	<0.0001	1.68 (1.27−1.84)	<0.0001

Model 1, adjusted for age, race, and gender; Model 2, adjusted for age, race, gender, and BMI. Hypertension (medical history or SBP ≥130 mmHg and DBP ≥80 mmHg); Diabetes (medical history or fasting glucose ≥ 126 mg/dL); Dyslipidemia (total cholesterol ≥ 200 mg/dL, triglycerides ≥ 150 mg/dL, LDL ≥ 100 mg/dL; or HDL < 40 mg/dL); Homocysteinemia (>13 µmol/L); Systemic inflammation (IL-6 > 17.5 pg/mL or CRP > 3.5 mg/dL); Arterial FMD (<8.0%); Arterial stiffness (>10.1 ms^−1^); Arteriolar FID (<33.8%); One-Carbon metabolism factors (folate < 16.5 ng/mL or B12 < 364 ng/mL); Inflammatory gene methylation score (<43.2%).

## Data Availability

All results from the current study are available in the article.

## Supplementary Materials

The following supporting information can be downloaded at: https://www.mdpi.com/article/10.3390/biomedicines10081954/s1. Table S1. Sequences of primers used for real-time PCR. Table S2. Percentage of DNA methylation of inflammatory genes in VAT.
