# Supplementary material for: DNA Hypomethylation as a Potential Link between Excessive Alcohol Intake and Cardiometabolic Dysfunction in Morbidly Obese Adults
Source: Biomedicines. 2022 Aug 12;10(8):1954. doi: 10.3390/biomedicines10081954 (PMC9406007; doi:10.3390/biomedicines10081954)
Supplement: Supplementary file 1 [file biomedicines-10-01954-s001.zip › biomedicines-1839504-supplementary.pdf]

Supplementary Table S1. Sequences of primers used for real-time PCR

Gene name	RefSeq Accession Number		Primer sequence	Tm	Amplicon size
CXCL1	NM_001511.4	Fw	ACTCTACCTGCACACTGTCC	59.03	219
		Rv	TCCCCTGCCTTCACAATGAT	59.00	
CXCR2	NM_001168298.2	Fw	TGGTGGTGAGTGCCTGTAAT	58.94	231
		Rv	GCTGCCATACTGTCTTCTGC	58.99	
HDAC5	NM_001382393.1	Fw	CCGGGTTTGATGCTGTTGAA	59.04	159
		Rv	CATCACAGATGGCGGTCAAG	58.99	
IGFBP3	NM_000598.5	Fw	GGGTAGGAGGGACAGAGAGA	59.07	247
		Rv	CAGCAAGCCATTCCTCCTTC	58.90	
IL12RB2	NM_001258214.1	Fw	CATAAACGCACCTCACCTCG	59.00	229
		Rv	ACTCGCTTCTCAGTTCAGCT	59.03	
IL1R1	NM_000877.4	Fw	GGGACTTTACACAGGGACCA	58.94	213
		Rv	GCAACGCCATAAGACAGGAG	58.99	
IL7	NM_000880.4	Fw	GGTGATTCGGAAATTCGCGA	59.00	158
		Rv	CGCCAGCAGTGTACTTTCAG	59.21	
IL12A	NM_000882.4	Fw	TCAGAATTCGGGCAGTGACT	59.02	163
		Rv	AGTCCCATCCTTCTTTCCCC	58.70	
IL17RA	NM_001289905.2	Fw	TGTTCCCATCTGCTCCCATT	59.00	183
		Rv	TTGTGCAGGGTCAATTGTGG	58.96	
MYD88	NM_001172566.2	Fw	CAGGAACAGCTAGGTGGGAA	59.02	240
		Rv	GGGAAGGAGAGAGGGAGAGA	59.07	
NFATC3	NM_004555.4	Fw	GATCCAGCGTCATTTCCACC	58.98	192
		Rv	TGGACATGAGAGCACACTGT	58.95	
NFκB	NM_001165412.2	Fw	AATGGTGGAGTCTGGGAAGG	59.00	226
		Rv	TCTGACGTTTCCTCTGCACT	58.96	
NFKBIB	NM_001243116.2	Fw	ATACCCCTGTCGCCTTGTAC	59.17	188
		Rv	CGGTTTGTCAAGGTCAGCTC	59.13	
SMAD3	NM_001145102.2	Fw	CTCTGGGTGCTTGGGAACTA	59.02	165
		Rv	ATCCAAATGCAGCCAAACGT	59.03	
TGFBR2	NM_001024847.3	Fw	AAGGAAGGGACCCATGACAG	59.00	179
		Rv	ATGGCCAGAAGAGAAGTGCT	59.01	
TLR5	NM_003268.6	Fw	CTGACTCGTTCTCTGGGGTT	59.03	156
		Rv	CCCGGAACTTTGTGACTGTG	59.06	
TNFRSF8	NM_001243.5	Fw	CTGTTTACTCATCGGGCAGC	58.99	197
		Rv	AGACACCCACTCCATCCTTG	59.01	
TRAF6	NM_004620.4	Fw	TCTTGTGACAACTGTGCTGC	58.98	165
		Rv	GCATGGAATTGGGGCTGTAG	58.96	

Supplementary Table S2. Percentage of DNA methylation of inflammatory genes in VAT

Gene	% Methylated ± SD			
	No/Mild	Moderate	Heavy	P-value
ABCF1	36.0% ± 45.4%	30.1% ± 13.1%	41.3% ± 22.4%	NS
ADA	51.7% ± 50.4%	26.7% ± 40.8%	21.6% ± 37.5%	NS
ATF2	54.9% ± 48.0%	41.7% ± 49.2%	33.3% ± 57.7%	NS
BCL10	40.0% ± 50.7%	16.8% ± 40.7%	27.4% ± 37.3%	NS
BCL3	30.1% ± 44.9%	27.2% ± 13.2%	32.2% ± 11.1%	NS
BCL6	33.0% ± 43.2%	42.1% ± 47.8%	17.3% ± 29.6%	NS
BLM	53.2% ± 51.5%	16.7% ± 40.8%	47.1% ± 14.1%	NS
CCL25	51.0% ± 45.6%	42.0% ± 48.8%	19.7% ± 33.5%	NS
CD274	52.3% ± 46.7%	61.5% ± 37.6%	33.3% ± 28.9%	NS
CD276	47.4% ± 46.9%	29.9% ± 20.0%	16.7% ± 28.9%	NS
CD40	52.0% ± 45.7%	58.0% ± 46.6%	16.7% ± 28.9%	NS
CD47	20.7% ± 41.1%	9.4% ± 20.0%	47.7% ± 46.6%	NS
CD7	72.2% ± 43.9%	47.2% ± 37.8%	76.8% ± 16.5%	NS
CD8A	52.3% ± 45.9%	14.8% ± 36.1%	47.1% ± 44.2%	NS
CEBPB	38.3% ± 44.9%	25.0% ± 41.8%	16.7% ± 28.9%	NS
CSF1	53.3% ± 46.7%	16.7% ± 40.8%	26.7% ± 18.9%	NS
CXCL1	57.5% ± 40.8%	9.2% ± 20.0%	9.8% ± 14.8%	<0.0005
CXCL12	46.7% ± 51.4%	21.1% ± 33.1%	27.0% ± 46.7%	NS
CXCL14	37.3% ± 39.2%	23.8% ± 38.9%	28.1% ± 46.6%	NS
CXCL2	47.8% ± 40.2%	17.3% ± 40.5%	35.2% ± 30.1%	NS
CXCL3	53.8% ± 48.9%	28.6% ± 45.2%	19.0% ± 32.8%	NS
CXCL5	37.9% ± 44.2%	28.1% ± 18.6%	13.0% ± 3.2%	NS
CXCL6	49.4% ± 46.5%	33.1% ± 21.3%	30.5% ± 20.4%	NS
CXCR4	55.4% ± 25.5%	45.3% ± 11.6%	29.5% ± 11.1%	<0.0005
DPP4	32.8% ± 44.4%	30.1% ± 46.8%	27.6% ± 35.4%	NS
EGR1	42.2% ± 44.3%	16.9% ± 36.9%	20.4% ± 21.2%	NS
FADD	37.8% ± 48.0%	11.1% ± 20.2%	30.4% ± 52.3%	NS
FOS	23.8% ± 40.3%	29.8% ± 45.9%	31.0% ± 21.0%	NS
FOXJ1	67.0% ± 47.8%	47.3% ± 52.1%	46.7% ± 44.2%	NS
FOXP3	70.3% ± 28.0%	76.1% ± 38.6%	92.7% ± 4.4%	NS
GATA3	29.0% ± 37.9%	32.5% ± 50.1%	33.2% ± 57.2%	NS
HDAC4	85.9% ± 17.6%	68.8% ± 14.9%	15.5% ± 3.8%	<0.0005
HMOX1	46.2% ± 51.1%	39.8% ± 46.6%	30.0% ± 10.0%	NS
ICOSLG	36.9% ± 47.8%	33.5% ± 50.2%	33.3% ± 57.7%	NS
IGFBP3	93.9% ± 34.1%	89.1% ± 22.8%	48.9% ± 13.8%	<0.0005
IL10RA	35.1% ± 41.9%	38.1% ± 43.6%	19.9% ± 31.0%	NS
IL10RB	27.3% ± 39.8%	24.1% ± 38.6%	20.2% ± 10.2%	NS
IL12A	41.9% ± 44.7%	14.5% ± 8.9%	10.5% ± 12.1%	<0.0005
IL12RB2	84.4% ± 15.7%	22.3% ± 9.5%	17.2% ± 11.5%	<0.0005
IL13	37.3% ± 38.1%	58.4% ± 46.5%	33.3% ± 57.7%	NS
IL13RA1	50.0% ± 29.2%	37.6% ± 34.0%	33.2% ± 14.7%	NS
IL15	69.9% ± 40.1%	44.1% ± 49.6%	33.0% ± 57.1%	NS
IL15RA	39.2% ± 45.5%	22.2% ± 36.0%	30.2% ± 10.2%	NS
IL17C	42.3% ± 41.0%	25.6% ± 38.4%	40.4% ± 15.5%	NS

IL17RA	37.3% ± 35.9%	12.8% ± 3.5%	9.2% ± 6.3%	<0.0005
IL1R1	88.3% ± 18.9%	52.3% ± 19.1%	33.4% ± 17.4%	<0.0005
IL36B	80.8% ± 35.6%	81.9% ± 48.9%	99.7% ± 35.3%	NS
IL4R	31.6% ± 32.9%	26.2% ± 42.1%	50.0% ± 49.5%	NS
IL6R	39.4% ± 44.2%	30.2% ± 36.8%	32.3% ± 36.9%	NS
IL6ST	45.5% ± 41.2%	22.5% ± 34.9%	17.4% ± 30.0%	NS
IL7	93.0% ± 19.5%	86.3% ± 18.7%	22.8% ± 8.1%	<0.0005
INHA	27.3% ± 39.0%	36.7% ± 49.2%	20.5% ± 35.2%	NS
INHBA	37.2% ± 41.4%	54.6% ± 45.0%	22.0% ± 37.1%	NS
IRF1	28.9% ± 38.1%	19.4% ± 39.9%	20.9% ± 36.0%	NS
JUN	53.5% ± 46.2%	16.7% ± 40.8%	38.0% ± 32.9%	NS
LAG3	24.5% ± 31.5%	19.2% ± 39.1%	35.8% ± 55.7%	NS
LCK	36.2% ± 41.0%	39.5% ± 46.1%	21.7% ± 34.6%	NS
LTB	41.4% ± 35.2%	18.9% ± 29.2%	2.3% ± 4.0%	NS
LTB4R	32.3% ± 32.6%	41.9% ± 44.7%	50.0% ± 49.7%	NS
MALT1	41.4% ± 39.4%	30.4% ± 31.1%	27.0% ± 43.5%	NS
MAP3K7	57.3% ± 38.9%	20.6% ± 33.6%	48.3% ± 42.9%	NS
MAPK14	53.3% ± 42.5%	39.5% ± 36.1%	31.6% ± 27.5%	NS
MICB	54.1% ± 40.0%	45.4% ± 49.8%	68.4% ± 27.0%	NS
MIF	45.1% ± 36.0%	17.5% ± 29.5%	30.2% ± 17.1%	NS
MYD88	69.5% ± 33.4%	25.4% ± 11.6%	25.5% ± 5.5%	<0.0005
NCK1	59.0% ± 44.7%	68.7% ± 37.9%	54.3% ± 47.4%	NS
NFATC3	47.4% ± 13.8%	44.7% ± 16.5%	32.1% ± 10.7%	<0.0005
NFKB1	94.5% ± 14.1%	53.7% ± 27.2%	55.9% ± 19.5%	<0.0005
NFKBIB	54.8% ± 41.9%	20.0% ± 12.4%	12.7% ± 21.4%	<0.0005
NOD1	47.8% ± 42.4%	49.6% ± 41.5%	77.2% ± 24.2%	NS
NR3C1	36.3% ± 41.6%	52.4% ± 41.6%	15.6% ± 17.8%	NS
PAX1	65.0% ± 45.4%	51.2% ± 44.8%	39.9% ± 40.2%	NS
PF4	50.0% ± 31.8%	50.6% ± 44.6%	66.8% ± 29.0%	NS
PTGS2	35.4% ± 40.5%	42.9% ± 36.8%	40.0% ± 30.0%	NS
RELA	25.1% ± 30.9%	28.3% ± 40.0%	20.1% ± 16.2%	NS
RIPK2	52.5% ± 40.5%	15.1% ± 36.8%	26.1% ± 42.6%	NS
RUNX1	47.6% ± 42.7%	27.7% ± 44.0%	42.1% ± 36.4%	NS
S1PR3	49.0% ± 44.6%	33.1% ± 51.3%	33.3% ± 57.7%	NS
SMAD3	95.2% ± 15.2%	69.1% ± 11.5%	57.5% ± 19.1%	<0.0005
SOCS1	44.5% ± 38.7%	31.7% ± 49.0%	50.0% ± 40.0%	NS
SOD1	50.6% ± 42.3%	46.2% ± 50.8%	33.3% ± 57.6%	NS
STAT1	59.5% ± 39.3%	21.6% ± 39.0%	38.4% ± 33.3%	NS
STAT5A	52.4% ± 45.5%	38.7% ± 42.5%	68.4% ± 9.8%	NS
TGFBR2	94.9% ± 24.4%	26.8% ± 19.6%	16.7% ± 11.5%	<0.0005
NELFCD	58.5% ± 44.5%	33.4% ± 51.6%	20.7% ± 34.2%	NS
THY1	63.3% ± 48.0%	41.7% ± 49.2%	66.7% ± 57.7%	NS
TIRAP	44.2% ± 43.6%	25.2% ± 41.2%	20.5% ± 35.4%	NS
TLR2	59.8% ± 45.6%	33.8% ± 51.2%	36.4% ± 35.3%	NS
TLR5	35.7% ± 16.6%	17.9% ± 7.6%	5.6% ± 4.1%	<0.0005
TNFRSF1B	31.1% ± 9.3%	25.1% ± 7.5%	22.6% ± 5.3%	<0.0005
TOLLIP	50.8% ± 49.8%	33.3% ± 51.6%	35.2% ± 54.6%	NS
TRAF2	50.6% ± 49.2%	27.4% ± 43.9%	17.3% ± 28.3%	NS

TRAF6	80.0% ± 25.2%	81.6% ± 17.5%	50.5% ± 22.3%	<0.0005
TYK2	70.0% ± 45.5%	83.2% ± 40.8%	91.0% ± 20.0%	NS

NS, not significant
